# Multiple Roles of the Small GTPase Rab7

**DOI:** 10.3390/cells5030034

**Published:** 2016-08-18

**Authors:** Flora Guerra, Cecilia Bucci

**Affiliations:** Department of Biological and Environmental Sciences and Technologies (DiSTeBA), Università del Salento, Via Provinciale Lecce-Monteroni n. 165, 73100 Lecce, Italy; guerraflora@gmail.com

**Keywords:** Rab7, endocytosis, vesicular transport, membrane traffic, lysosome, autophagy, mitophagy, lipophagy, apoptosis, intermediate filaments

## Abstract

Rab7 is a small GTPase that belongs to the Rab family and controls transport to late endocytic compartments such as late endosomes and lysosomes. The mechanism of action of Rab7 in the late endocytic pathway has been extensively studied. Rab7 is fundamental for lysosomal biogenesis, positioning and functions, and for trafficking and degradation of several signaling receptors, thus also having implications on signal transduction. Several Rab7 interacting proteins have being identified leading to the discovery of a number of different important functions, beside its established role in endocytosis. Furthermore, Rab7 has specific functions in neurons. This review highlights and discusses the role and the importance of Rab7 on different cellular pathways and processes.

## 1. Introduction

Rab proteins belong to the large superfamily of Ras-like GTPases, are conserved from yeast to mammals and have a crucial role in membrane trafficking. In particular, they are important for vesicle formation, transport and fusion but also for cargo selection and sorting, thus being fundamental for vesicular traffic in endocytosis, exocytosis and autophagy [1,2,3,4]. As all GTPases, Rab proteins are characterized by a cyclical mechanism of activation and inactivation depending on GTP binding and hydrolysis. The shuttling between the inactive state (GDP-bound) and active state (GTP-bound) and vice versa of a Rab protein is a process in which ancillary proteins are necessary. Indeed, guanine nucleotide exchange factors (GEFs) stimulate GDP dissociation to allow its replacement by GTP, while GTPase-activating proteins (GAPs) are essential to prompt GTP hydrolysis. Newly synthesized Rab proteins are recognized by REP (Rab escort protein) and presented to the prenylation enzyme RabGGT (Rab geranylgeranyl transferase) in order to be geranyl-geranylated and thus able to be anchored to membranes. In the cytoplasm, GDI (GDP Dissociation Inhibitor) is associated with inactive GDP-bound geranyl-geranylated Rab proteins, while GDF (GDI displacement factor) is required for GDI displacement and recruitment of Rabs to membranes where GEFs stimulate nucleotide exchange leading to Rab activation [5,6]. 

Each Rab protein localizes to specific intracellular compartments and, in its GTP-bound form, interacts with a number of different proteins, defined effectors, which regulate several downstream functions [7]. For instance, Rab5 is present on early endosomes and, through the recruitment of effector proteins, regulates several events that include cargo selection of forming endocytic vesicles, generation of phosphatidylinositol-3-posphate lipid (PI3P) on early endosomes, early endosome homotypic fusion, motility of early endosomes on actin and microtubules tracks and activation of signaling pathways at the early endosomal level [8,9,10,11,12,13]. At variance, Rab4, also localized to early endosomes, regulates the exit of constitutive recycling cargoes from this compartment directly back to the plasma membrane as well as to recycling endosomes, also called recycling compartment, [14,15], where Rab11 directs recycling to the plasma membrane [16]. Thus, each Rab protein regulates a specific step of vesicular trafficking. 

In mammals, there are two Rab7 proteins, Rab7a and Rab7b that, however, cannot be defined Rab isoforms as their identity is limited to 50% and they do not share the RabF and RabSF motifs [17,18]. In fact, they are differently localized and control different steps of transport. Rab7a is localized mainly to late endosomes and regulates transport to late endocytic compartments thus transport from early endosomes to late endosomes and from late endosomes to lysosomes [19]. In contrast, Rab7b controls endosomes to Golgi transport, being localized both to the Trans Golgi Network (TGN) and to late endosomes [20,21]. This review will focus on the Rab7a isoform, hereafter referred as Rab7.

**Figure 1:**
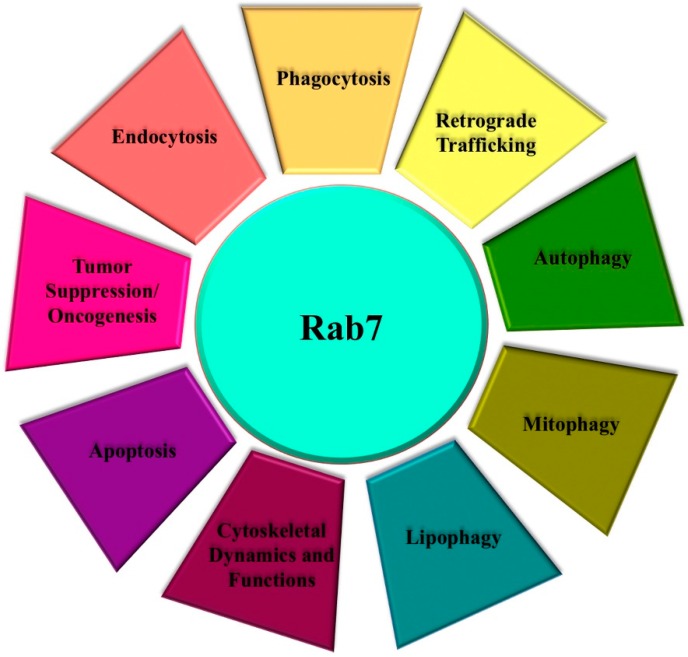
Multiple roles of Rab7. Rab7 has different roles in several crucial cellular functions.

Rab7 controls maturation of early endosomes in late endosomes, transport from late endosomes to lysosomes, biogenesis of lysosomes and clustering and fusion of late endosomes and lysosomes in the perinuclear region [19,22,23,24] (Figure 1). 

Rab7 is one of the most studied Rab proteins and several data in the literature indicate that this GTPase, controlling maturation of endosomes and transport to lysosomes, has a role in several physiological process, such as apoptosis [25], neurotrophin trafficking and signaling, neurite outgrowth [26,27], phagocytosis [28], autophagy and mitophagy [29,30] (Figure 1). Furthermore, Rab7 dysfunction leads to disease as it has been demonstrated a role of Rab7 as a tumor suppressor [31,32] and mutations in Rab7 cause the Charcot-Marie-Tooth type 2B (CMT2B) peripheral neuropathy [33,34].

In light of the polyhedric role of Rab7 in the economy of the cell, the aim of this review is to give a detailed overview of the impact of the endocytic Rab7 activity on different cellular processes. We will focus on mammalian Rab7 although we will occasionally also refer to the role of Rab7 in other organisms when it helps to clarify Rab7 functions and/or mechanisms of action.

## 2. Rab7 and Endocytosis

The internalization of solutes, macromolecules, fluids, plasma membrane component and particles occurs in cells through a process known as endocytosis and characterized by modifications of the shape of the plasma membrane to form vesicles and vacuoles through membrane fission events [35]. Internalization of material is achieved via clathrin-mediated pathway or several other clathrin-independent pathways, including caveolar- and ARF6-dependent pathways [36]. The incoming soluble or plasma membrane cargoes are addressed to early endosomes, also called sorting endosomes as they represent the main station of the endocytic pathway, being the first compartment to receive and sort incoming cargoes [37]. From sorting endosomes, cargoes follow different fates: they can be recycled to the plasma membranes, directly or via perinuclear recycling endosomes or, alternatively, can be delivered to late endosomes and lysosomes for degradation [38]. The main examples of recycled and degraded cargoes are represented by transferrin receptor (TfR) and epidermal growth factor receptor (EGFR), respectively [35]. Delivery of endocytosed material to lysosomes for degradation occurs after progressive acidification of endosomal organelles, formation of multivesicular bodies (MVBs) and late endosomes, recruitment of lysosomal hydrolases from Golgi, positioning of late endosomes in the perinuclear region and sorting of proteins destined for degradation [39]. 

### 2.1. Role of Rab7 in the Late Endocytic Pathway

**Figure 2:**
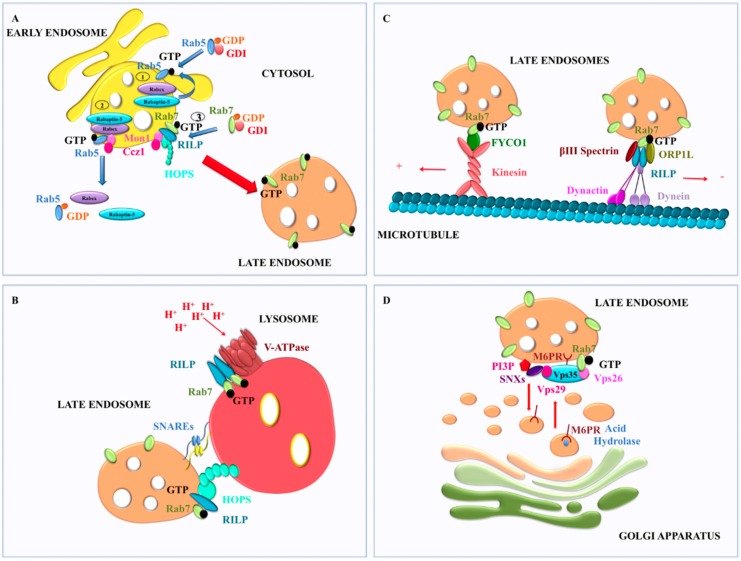
Rab7 in the late endocytic pathway and in retromer regulation: (**A**) sequential and dynamic cooperation between Rab5 and Rab7 to determine the Rab5 to Rab7 switch; (**B**) RILP interaction with HOPs complex for late endosome-lysosome fusion and Rab7–RILP interaction to regulate assembly and function of the V-ATPase for acidification; (**C**) movement of late endosomes on microtubules is determined by interaction of Rab7 with RILP and FYCO1 for minus-end and plus-end direction, respectively; and (**D**) retrograde transport of transmembrane cargo from endosomes to the TGN is regulated by interaction between Rab7 and subunits of the retromer complex.

Rab7 is directly or indirectly involved in each event that occurs between early endosomes and lysosomes. The endocytic pathway is characterized by a complex and dynamic maturation of endosomes that leads to the formation of late endosomes from early endosomes after the generation of Rab7 domains [40,41]. Rab7 is normally present on late endosomes and the acquisition of Rab7 on late endosomes is accompanied by the loss of Rab5, which is instead a marker of early sorting endosomes. This process is known as the Rab5 to Rab7 switch and consists in the sequential and dynamic cooperation between Rab5 and Rab7 that determines initially the recruitment of Rab5 to early sorting endosomes and, subsequently, the recruitment of Rab7 and the loss of Rab5 at late endosomes [40,41,42]. Recruitment of Rab5 to early endosomes is accompanied by Rab5 activation mediated by Rabex-5 [43,44], a Rab5 GEF whose activity is promoted by the Rab5 effector Rabaptin-5. Rabex-5, Rabaptin-5 and GTP-bound Rab5 form a complex [43] that is required to establish a feedback loop, whereby Rab5–GTP promotes further Rab5 binding [45] leading to a rapid recruitment on Rab5-positive organelles of numerous Rab5 effectors [16,46]. Both the inhibition of the feedback loop for Rab5 binding and a GAP activity to stimulate Rab5 GTP hydrolysis become essential to remove Rab5 and to replace it with Rab7. SAND-1/Mon1 and Ccz1, two factors recruited from the cytosol to endosomal membranes, have a key role in the Rab5 to Rab7 switch. In fact, in *Caenorhabditis elegans*, it has been demonstrated that SAND-1/Mon1 is able to interrupt the positive feedback loop of Rab5 activation by displacing Rabex-5 [42], and that the complex SAND-1/Mon1 and Ccz1 interacts with Rab5–GTP, thus being a Rab5 effector, but it is also able to recruit and activate Rab7 [47]. Furthermore, direct Rab7 GEF activity of the Mon1–Ccz1 complex was demonstrated in yeast using the purified complex [48,49]. These data were further confirmed by the finding that Rab7 is activated by the Mon1–Ccz1 complex at the level of late endosomes and dissociates from lysosomes in mammalian cells [50] (Figure 2A).

The Rab5/Rab7 switch is also accompanied by changes in the tethering and fusion machinery in order for late endosomes to acquire the ability to recognize and fuse with other late endosomes and with lysosomes. Tethering is an essential event for fusion allowing organelles to contact each other [38]. The CORVET (class C CORe Vacuole/Endosome Tethering) and HOPS (HOmotypic fusion and Protein Sorting) complexes are an important part of the tethering and fusion machinery for early and late endosomes, respectively, and they were first identified and better characterized in yeast [51]. In fact, in yeast, the HOPS complex has an elongated seahorse-like structure, needed to bridge membranes, with a large head domain and a tail domain, [52]. Both ends of this structure are able to bind the yeast Rab7 counterpart, Ypt7, through the Vps41 subunit in the head and the Vps39 subunit in the tail [52]. The large head domain also contains Vps33, which it is able to bind the SNARE (Soluble N-ethylmaleimide-sensitive fusion protein-Attachment protein REceptor) complex [53,54,55,56]. Thus, in yeast, the HOPS complex forms a bridge between Ypt7-positive membranes but also recruits SNAREs at fusion sites [52,55,57]. SNARE proteins are the core machinery for fusion and, undergoing a folding process, assemble in a zipper-like fashion bringing together two membranes and providing the energy for membrane fusion [58].

In mammalian cells, the molecular mechanisms of action of the HOPS complex are not completely defined yet but they seem to differ from yeast [59,60,61,62]. In fact, the small GTPase Arl8b, but not Rab7, is required for recruitment of human Vps41 on lysosomal membranes and for subsequent assembly of the core HOPS subunits [62]. In addition, an amino-terminal domain of the Rab Interacting Lysosomal Protein (RILP), an effector of Rab7 not present in yeast, interacts with the carboxyl-terminal region of VPS41 of the HOPS complex and recruits HOPS subunits to the late endosomal compartment in a Rab7-independent manner [61]. Furthermore, RILP, interacting with both Rab7 and ORP1L (oxysterol-binding protein-related protein 1 L), binds the tethering HOPS complex and the p150^Glued^ subunit of the dynein motor, thus efficiently coupling the timing of microtubule minus-end transport and of fusion [60] (Figure 2B). Therefore, the role of Rab7 in the recruitment and functions of HOPS is still unclear in mammalian cells.

Motility of endosomes and lysosomes depends on dynein and kinesin motors that provide opposing forces to move endosome in opposite direction [63,64] (Figure 2C). The movement of late endosomes on microtubules that brings them near lysosomes is minus-end-directed and is mainly regulated by dynein [65,66]. In this process, Rab7 in its GTP-bound form mediates attachment of late endosomes to dynein–dynactin complex through the recruitment of RILP [67,68]. In fact, upon membrane anchoring and activation, Rab7 interacts simultaneously with ORP1L and with RILP [69]. Next, the Rab7–RILP complex is transferred by ORP1L to βIII spectrin for starting the translocation to microtubules thanks to interaction between βIII spectrin and dynein [69]. Furthermore, recent evidence demonstrated that interaction between Rab7 and FYVE and coiled-coil domain containing protein (FYCO) 1 is able to regulate plus-end directed motility and, hence, transport of late endosomes to the periphery, probably by means of kinesin motors [70]. Thus, Rab7 is fundamental for movement of Rab7-positive vesicles and organelles on microtubules both towards plus and minus ends.

Once late endosomes are translocated near the MTOC, fusion between late endosomes and lysosomes occurs. Today, there are at least three alternative hypotheses about the mixing between the late endosome and lysosome content. The first proposes the occurrence of vesicular traffic between the two organelles; the second envisages continuous fusion and fission events between endosomes and lysosomes accordingly to the “kiss an run” model; and the third predicts direct fusions between late endosome and lysosome to form a hybrid organelle, called endolysosome, in which degradation takes place, and, after this fusion events, lysosomes are reformed from the endolysosome, becoming storage organelles for lysosomal hydrolases and membrane components that can be reutilized [35,38,71]. The latter hypothesis is considered an extension of the “kiss and run” model.

Which are the steps of transport regulated by Rab7 in the endocytic pathway? Some data indicate that it controls transport from early endosomes to late endosomes [72,73]. In fact, it was demonstrated that trafficking of the VSV (vesicular stomatitis virus) G protein from early endosomes to late endosomes was blocked by constitutive expression of the dominant-negative Rab7 T22N and N125I mutant proteins, without affecting its internalization from the surface [72]. Furthermore, constitutive expression of these dominant negative Rab7 mutant proteins caused accumulation of cathepsin D and of the cation-independent mannose-6-phosphate receptor (CI-M6PR) in early endocytic compartments [73]. However, these studies were conducted in stable cell lines that had to adapt to the constitutive presence of the Rab7 mutant proteins. Other reports, instead, indicate that Rab7 regulates transport from late endosomes to lysosomes. Indeed, it was demonstrated that Rab7 resides in a compartment connected with lysosomes and that transient expression of Rab7 dominant negative mutants affect LDL (low-density lipoproteins) degradation [74,75]. In addition, Rab7 is fundamental for the biogenesis and functional maintenance of lysosomes [19]. Furthermore, expression of Rab7 dominant negative mutants causes dispersal of late endosomes with disappearing of the perinuclear late endosomal and lysosomal cluster and block of cargo trafficking to the lysosomes [19]. At variance, expression of Rab7 constitutively active Q67L mutant or overexpression of the wild-type protein causes the formation of large endocytic structures densely packed in the perinuclear region [19]. In addition, it was demonstrated that Rab7 function is exerted downstream of MVBs formation [76,77]. 

Interestingly, in cells expressing the Rab7 dominant negative T22N mutant lysosome acidification is severely perturbed [19], suggesting problems with the regulation of vacuolar ATPase (V-ATPase), the enzyme responsible for the low intralysosomal pH required for functioning of lysosomal acid hydrolases [78,79,80]. In fact, it was recently demonstrated that Rab7, through its effector RILP, regulates lysosomal pH by controlling assembly and function of the V-ATPase on Rab7-positive organelles through interaction of RILP with the V1G1 subunit [81,82] (Figure 2B). Furthermore, it has been recently demonstrated that regulation of lysosomal pH controlled by Rab7 and RILP depends also on lysosomal positioning [83]. Therefore, Rab7 regulates not only late endosomal trafficking but also late endosomal and lysosomal pH. 

### 2.2. Role of Rab7 in Phagocytosis

The aim of phagocytosis is the internalization of particulate matter such as microorganisms, apoptotic cell bodies and cell debris. In mammals phagocytosis is a crucial step of innate and adaptive immunity, aimed to entrap, kill and degrade microorganisms in order to present their antigens to lymphoid cells [84]. To this end, leukocytes are attracted and stimulated by the activated phagocytes through secretion of cytokines. In addition to immune responsiveness, phagocytic cells contribute to tissue homeostasis and remodeling by removing apoptotic bodies [85]. The nascent phagosome derives from the plasma membrane and initially it does not possess microbicidal and degradative properties. These capabilities are acquired subsequently, in a slow, gradual and complex process, called phagosome maturation, characterized by a sequence of membrane fusion and fission events, coupled to the acquisition and activation of an arsenal of oxidative, acidifying and hydrolytic enzymes [86]. After maturation, the phagosome becomes a phagolysosome. Similar to what occurs in the endocytic pathway, maturation of a phagosome proceed gradually and consists in early, late and lysosome-interacting stages. The presence of Rab5 is characteristic of early phagosomes and is required for the transition to the late phagosomal stage with the acquisition of Rab7 domains [87]. Rab7 is essential for the fusion of phagosomes with late endosomes and lysosomes, for functional phagosomal acidification and for centripetal displacement of phagosomes [28,87]. During maturation, phagosomal movement from the cell periphery to the MTOC and the projection of tubes from phagosomes facilitate their fusion with lysosomes [28,88]. This displacement is due to RILP, recruited to phagosomes by active Rab7, and ORP1L, which allow phagosomes to bind the dynein–dynactin microtubule motor and move towards the MTOC [88]. Thus, Rab7 is a key protein for the biogenesis of phagolysosomes being important for the acquisition of microbicidal and degradative properties. Notably, subversion of phagosomal maturation by pathogens is often accomplished through alteration of Rab7 and/or Rab7 effector functions [89,90].

### 2.3. Role of Rab7 in Retromer Regulation

Secretory and endocytic pathway are connected by transport from the trans-Golgi network (TGN) to endosomes in order to deliver newly synthesized acidic hydrolases to the endocytic pathway and by transport from endosomes to the TGN in order to recycle receptors. The evidence of Rab7 implication in each step of endosomal trafficking emerges from its role also in the retrograde transport between endosome and the Golgi apparatus [91] (Figure 2D). In fact, retrograde transport of transmembrane cargo from endosomes to the TGN is regulated by a phylogenetically conserved multisubunit complex, known as the retromer [91,92,93]. This complex is constituted by two distinct subcomplexes: (i) a heterotrimer cargo recognition complex, composed of Vps26, Vps29, and Vps35; and (ii) a sorting nexin (SNX) complex consisting of a heterodimer or homodimer of SNX1 or SNX2 combined with SNX5 or SNX6 [94,95,96,97,98]. 

The CI-M6PR is the best-characterized cargo of the mammalian retromer [99]. The CI-M6PR binds newly synthesized acid hydrolases at the TGN and carries them to endosomes, where the hydrolases are released to be transported to lysosomes. The essential role of retromer is the retrieval of the unoccupied receptors to the TGN, where they are engaged in further cycles of acid hydrolase sorting. Depletion of retromer subunits by RNA interference prevents this retrieval, leading to rerouting of the receptors to lysosomes and to leakage of newly synthesized acid hydrolases into the extracellular medium [98,100,101,102]. Rab7 interacts directly with the cargo recognition complex of the retromer and, while Rab5 interacts with the phosphatidyinositol-3 kinase (PI3K) and recruits SNX1/2 to early endosomal membranes, Rab7 recruits the retromer core complex on late endosomal membranes through direct interaction with Vps26 [103] (Figure 2D). The cargo recognition complex localizes to endosomal domains that contain Rab7 and expression of a dominant-negative Rab7 mutant or Rab7 silencing cause dissociation of the cargo recognition complex from membranes, inhibition of CI-M6PR retrograde transport, and missorting of the acid hydrolases [103]. Rab7 interacts directly also with Vps35 to recruit retromer to late endosomes and interaction between Vps26 and Vps35 is essential to increase the affinity between the cargo recognition complex of the retromer and activated Rab7 [104] (Figure 2D). Altogether these data indicate that Rab7 is fundamental for the recruitment and functioning of the retromer at late endosomes. In agreement with the studies in mammalian cells [103,105], it was demonstrated that in yeast Ypt7 can coordinate retromer functions on endosomes [106,107]. In fact, retromer recycling of Vps10, a single-pass type-I transmembrane protein with a large luminal N-terminus that binds cargo molecules and a small cytosolic C-terminus that contains sorting signals for retrieval [108,109], requires active Ypt7 [110].

Interestingly, it was recently demonstrated that Parkin (a component of the E3 ubiquitin ligase complex whose dysfunction cause Parkinson’s disease) regulates Rab7 [111]. In fact, Rab7 undergoes Parkin-dependent ubiquitination on conserved K38 residue that enhances RILP–Rab7 binding thus regulating Rab7 activity. Loss of Parkin function determines impairment of the retromer pathway, decrease of endosomal tubulation and of membrane association of Vps35, and SNX1, coupled with increased exosome release [111]. Similar effects are detected upon expression of the Rab7 K38R mutant, thus suggesting that Parkin phenotype is at least in part dependent on Rab7 activity [111].

## 3. Rab7 and Autophagy

Autophagy (from Greek, meaning “self-eating”) is the major pathway responsible for maintaining cell homeostasis, being responsible for destruction of unnecessary or dysfunctional molecules and organelles [112,113]. Autophagy is induced by withdrawal of nutrients and various stress conditions, such as alterations in glucose metabolism [114,115], mitochondrial dysfunction and oxidative stress [116,117], and it is finalized to remove damaged macromolecules and organelles and/or to digest cell components in order to help the cell’s own maintenance [118,119]. Three kinds of autophagy have been described up to know: macroautophagy, microautophagy and chaperone-mediated autophagy that differ for the way cytosolic components are delivered to lysosomes for proteolytic degradation.

Autophagy was initially described as a degradative pathway in which double membrane compartments, defined autophagosomes, sequester damaged cargo for degradation through delivery to the acidic lysosomal compartment [120]. In general, the term “autophagy” refers to macroautophagy, a multistep process by which portions of cytoplasm and/or organelles are sequestered in a double or multimembrane structure, the autophagosome, and delivered to the lysosome for degradation. This pathway starts with growing of a membrane around cytosolic components to form initially the phagophore, also called isolation membrane. Subsequently, the phagofore, through a specific series of events, elongates, engulfing a portion of cytoplasm and, when the organelle is sealed to form a closed and double-membrane organelle that completely encapsulate its cargo, it becomes an autophagosome. At present, more than 32 autophagy-related genes (ATGs), identified in yeast and with homologs in mammals, have been implicated in the regulation of this autophagic pathway [121].

Macroautophagy may be induced by endogenous and exogenous specific stimuli, such as nutrient deprivation or treatment with rapamycin [122], which converge in inhibition of mammalian target of rapamycin complex 1 (mTORC1). mTORC1 is a leading actor of the survival pathway and it activates translation of proteins [123]. During nutrient withdrawal or after treatment with rapamycin, mTORC1 is inhibited, preventing protein translation and determining phagophore nucleation and formation through a Beclin-dependent mechanism [124,125]. Beclin is the master member of PI3K initiation complex that mainly induces production of PI3P and cleavage of microtubule-associated protein 1A/1B-light chain 3 (LC3) to form LC3-I which is successively conjugated to phosphatidylethanolamine to form LC3-II, recruited to autophagosomal membranes [126].

Newly formed autophagosomes undergo a complex series of sequential fusion events with elements of the endocytic pathway in order to finally mature into autolysosomes, organelles in which lysosomal hydrolases take care of cargo degradation [127]. In fact, autophagosomes fuse initially with early endosomes and form amphisomes, organelles that are still lacking lysosomal enzymes and lysosomal membrane proteins but that have the ability to fuse with late endosomes [127,128]. Then, lysosomal hydrolases and membrane proteins are acquired in subsequent fusions with late endosomes and lysosomes [127,128]. During maturation, autophagosomes are subjected to centripetal movement towards perinuclear region to cluster around the nucleus and undergo acidification. Autophagosomes are also classified as early autophagic vacuoles when containing morphologically intact cytoplasm and organelles, or late degradative autophagic vacuoles when containing partially degraded materials [127,129]. Therefore, the final aim of these multiple fusion events is to provide an acidic environment with digestive function called autolysosome [127,129,130].

### 3.1. Role of Rab7 in Macroautophagy: Involvement in Autophagosomal Maturation

**Figure 3:**
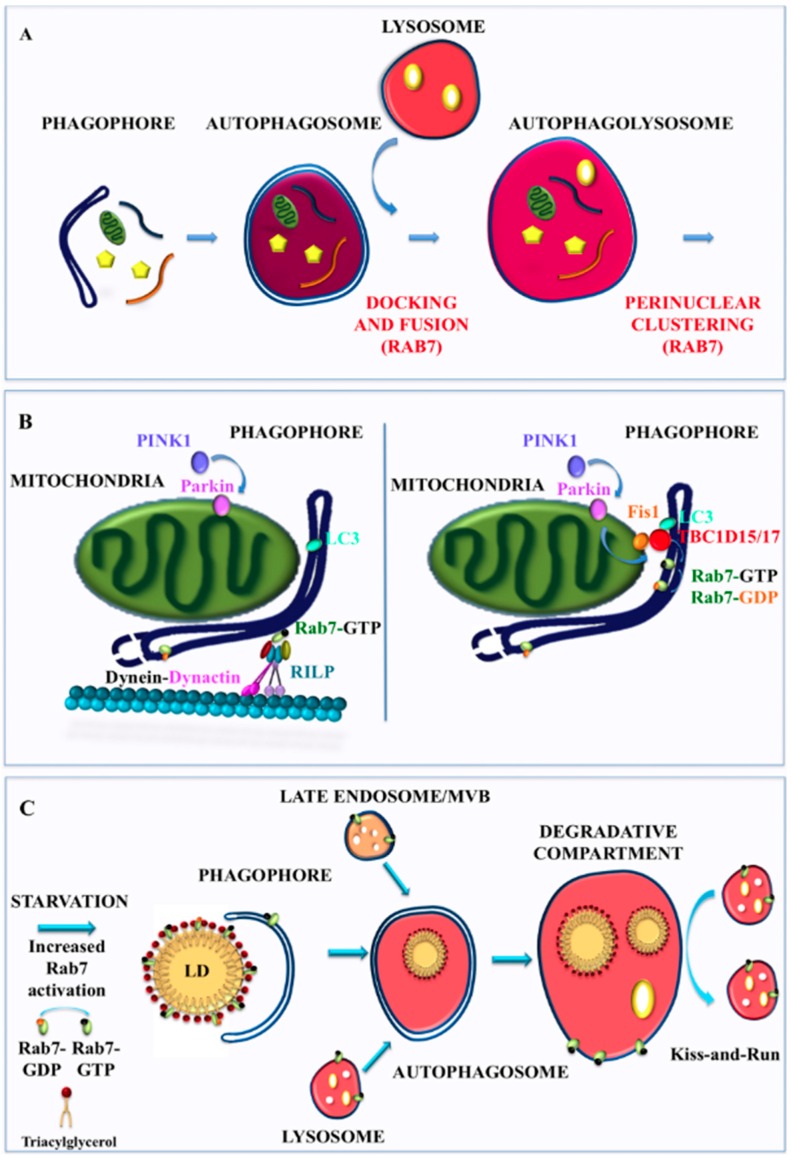
Rab7 in macroautophagy, mitophagy and lipophagy: (**A**) importance of Rab7 in docking, fusion and autophagosome clustering during macroautophagy; (**B**) Rab7 promotes the growth of the isolation membrane through microtubule associated trafficking but subsequently Rab7 inactivation is necessary for correct shaping of the autophagosomal membrane around mitochondria; and (**C**) increased Rab7 activation allows autophagy of LDs, a process named lipophagy. The autophagosome fuses with endocytic Rab7-positive degradative compartments to eventually form an autolysosome for lipid degradation.

The fusion events required for autophagosome maturation are governed by complex mechanisms involving multiple molecular machineries, including endosomal sorting complex required for transport (ESCRT), microtubules, SNAREs, the V-ATPase and Rab7 [131,132]. Similar to what occurs in the endocytic pathway, Rab7, in order to control fusion events, acts in concert with HOPS effectors and specific members of the SNARE family [133,134] (Figure 3A).

The fundamental importance of Rab7 in governing the mechanism of fusion was highlighted in cardiomyocytes Csn8 knockout [135]. Csn8 is a subunit of the COP9 signalosome for regulation of the ubiquitin proteasome system and extensive accumulation of autophagosomes in the absence of Csn8 was attributed to defective maturation and thus defective fusion of autophagosomes with late endosomes and lysosomes, accompanied by downregulation of Rab7 and leading to exacerbated necrosis or apoptosis [135,136]. The role of Rab7 in autophagosome late fusion events correlates with the activation of lysosomal functions during starvation. Indeed, it was observed that, after starvation or mTOR inhibition, Rab7 knockdown leads to the blockage of autophagosome fusions with late endosomes and lysosomes, inhibiting the physiological increase of lysosomal enzyme cathepsin B activity, similar to what occurs after treatments with chemicals that block fusion with lysosomes [137].

The key role of Rab7 in autophagosome maturation is emphasized by several studies in which it was demonstrated that Rab7 is essential for autophagosome clustering in the perinuclear region and for subsequent fusion with lysosomes [138,139] (Figure 3A). Increasing Rab7 labeling intensity was observed during vacuole maturation as Rab7-staining in limiting membrane of late autophagic vacuoles was much stronger than that of early vacuole, and Rab7 delivery to autophagosomes was detected before fusion with lysobisphosphatidic acid (LBPA) or Lamp-1 positive compartments [138,139]. Moreover, it was observed that structures Rab7-, LC3- and LBPA-positive became more evident in the perinuclear region with increasing starvation [138,139]. Rab7 silencing or expression of dominant negative Rab7 T22N mutant have highlighted that Rab7 depletion or Rab7 dysfunction do not affect initial maturation and fusion steps leading to the formation of morphologically identifiable late autophagic vacuoles, but determine accumulation of late autophagic vacuoles and inhibition of the formation of autolysosomes in light of the large, mainly perinuclear LC3- and LAMP1-positive structures absence [138,139]. Thus, Rab7 has a key role in the final maturation of autophagosomes to autolysosomes accomplished by fusion events with lysosomes [138].

A further common aspect of autophagosomes with endosomes is that the movement of the autophagosome from the periphery of the cell towards the perinuclear area is needed for its maturation [140]. Rab7 regulates autophagosome’s movement recruiting RILP that, in turn, recruits the dynein–dynactin motors that promote transport toward the minus-end of microtubules. Interestingly, alterations of autophagosome maturation were described in Purkinje neurons in which it was observed, under deprivation of trophic factor, occurrence of an autophagic associated cell death mechanism, due to the increase of autophagosome-to-lysosome fusion rate [141]. Furthermore, accumulation of autophagic organelles and cell death were inhibited by Insulin-like Growth Factor-1 (IGF-I) through regulation of the autophagosome to lysosome fusion mediated by Rab7 and RILP [142]. In fact, during prolonged tropic factor withdrawal, lower levels of active Rab7 bound to RILP are detected and autophagosomes accumulate in neurons [142]. However, IGF-I treatment is able to prevent deactivation of Rab7, restoring normal levels of the Rab7–RILP complex and thus the autophagic flux [142]. Therefore, IGF-I promotes Rab7–RILP binding under deprivation conditions and, as a result, the Rab7–RILP complex recruits the dynein–dynactin motor complex for transport of autophagosomes towards the MTOC and thus to lysosomes for fusion [142]. Autophagosomes are also able to move towards cell periphery on microtubules by plus-end transport using FYCO1. FYCO1 interacts with LC3, Rab7 and PI3P and it is localized on the external surface of autophagosomes as well as late endosomes and lysosomes to promote plus end–directed transport of these membranous compartments through binding of kinesin motors [70]. While in physiological conditions FYCO1 preferentially resides on the membranes of perinuclear endosomes in a conformation that prevents binding to kinesins, after amino acid starvation it binds to kinesin motors to redistribute them to pre-autophagosomal membranous compartments where autophagosome formation takes place. After autophagosomes are formed, FYCO1 competes with the RILP-dynein recruitment complex for binding to Rab7 regulating bidirectional transport of autophagosomes along microtubules [70]. In fact, in primary neurons, autophagosomes move bidirectionally along microtubules, driven by bound kinesin and dynein motors [143]. In particular, it was observed that during early stages of maturation, autophagosomes exhibit bidirectional motility in distal regions, but then escape from this distal pool to become fully acidified through fusion with late endosomes and lysosomes in the perinuclear region [143]. Thus, movement along the axon towards the cell soma, in a robust retrograde motility dynein-driven, accompanies autophagosome’s maturation [143]. During this minus-end movement, kinesin motors remain stably associated with autophagosomes [143]. Degradation of cargo occurs near the cell soma, the primary site of protein synthesis, probably to facilitate efficient recycling of amino acids and lipids, and then mature autolysosomes revert to bidirectional motility resembling that of lysosomes [143]. Altogether, these data indicate that Rab7 is fundamental for movement of autophagosomes on microtubules.

Upregulation of Rab7 improves the autophagic flux and the important implications of this concept have been underlined considering the observed decrease of autophagy in the age-related loss of cardioprotection [144]. Aldehyde dehydrogenase 2 (ALDH2) is abundantly expressed in heart and brain with pivotal role in aldehyde detoxification [145] and its dysfunction is associated with the process of aging and age-related cardiovascular diseases [146]. Importance of Rab7 in autophagic flux with implication in cardioprotection emerged from evidences indicating that ALDH2 induces upregulation of Rab7 increasing the autophagic flux under stress stimuli [144].

Notably, the attempts to define precisely events taking place during the last steps of autophagosome maturation demonstrated the existence of a negative feedback mechanism to reverse autophagy and restore lysosome homeostasis termed Autophagic Lysosome Reformation (ALR) [147]. The ALR mechanism is determined by re-activation of mTOR after 6 h of starvation and is regulated by Rab7. In fact, while after 4 h of nutrient deprivation essentially all lysosomes are consumed giving rise to few and large LAMP1-positive autolysosomes, after 12 h of starvation, the pool of lysosomes is restored in number and size [147]. This process is mediated by the formation of LAMP1-positive tubular structures coming from autolysosomes but devoid of the luminal content typical of these organelles [147]. These tubular structures are constituted by membrane components of lysosomes recycling from autolysosomes, are highly dynamic and undergo budding [147]. In fact, in these structures, lysosomal membrane proteins, such as LAMP1 and LAMP2, are present, but not autophagosomal membrane proteins, such as LC3 [147]. Furthermore, they are not acid and lack of capacity to process the substrates and have been called for these reasons, proto-lysosomes [147]. The key regulatory role of Rab7 in the formation of these structures was demonstrated by the fact that treatment with GTPγS, a non-hydrolyzable analog of GTP, completely inhibited ALR leaving only enlarged autolysosomes. Moreover, after 8 or 12 h of starvation, Rab7 was detected only in lysosomal fractions, but not in proto-lysosome and tubule fractions, suggesting that Rab7 must dissociate from tubules before reformation can proceed [147]. Indeed, overexpression of a constitutively active mutant of Rab7, permanently membrane-associated, abrogates ALR, resulting in enlarged and long-lasting autolysosomes [147]. Interestingly, treatment with rapamycin determines inhibition of ALR but also blocks Rab7 dissociation from autolysosomes, indicating that mTOR potentially regulates ALR through Rab7 [147,148]. Further confirmation of Rab7 role in ALR was obtained by treating cells with H_2_O_2_ to induce of autophagy [149]. Similar to starvation induction, Rab7 association to and dissociation from autolysosomes was observed at 4 h and 12 h after treatment, respectively [149]. 

### 3.2. Role of Rab7 in Mitophagy

In light of their importance in the life of eukaryotic cells, mitochondria undergo strict quality control check. Three pathways are implicated in preservation of a healthy mitochondrial population: (1) mitochondrial AAA protease complexes, localized in the in the inner membrane and implicated in the degradation of unfolded membrane proteins [150]; (2) the selective system for removal oxidized mitochondrial proteins through mitochondrial vesicles budding which sequester selected damaged cargos, and then deliver those mitochondrial components to lysosomal degradation, leaving the whole organelle intact [151]; and (3) the mechanism responsible for mitochondrial turnover and clearance of damaged or superfluous mitochondria, known as mitophagy, in which whole mitochondria are sequestered and delivered to lysosomes for hydrolytic degradation [152].

The different autophagy steps, such as the formation, elongation and closure of the isolation membrane, autophagosome formation and maturation, and degradation of cargo in autolysosomes, are required also for the clearance of a mitochondrion [153]. In addition, the core machinery constituted by ATGs genes is also essential for mitophagy [154]. Mitophagy in mammalian cell types may be cell type specific, as shown in the complete removal of mitochondria during erythrocyte maturation and in the selective destruction of sperm-derived mitochondria after oocyte fertilization [155,156,157]. 

The main actors that regulate mitophagy are represented by the mitochondrial protein kinase, PTEN-induced kinase 1 (PINK1), and the ubiquitin E3 ligase, Parkin [158,159]. In mammalian cells, activation of PINK1 in response to mitochondrial depolarization stimulates the recruitment of Parkin, a cytosolic protein, to depolarized mitochondria [160,161,162,163]. In particular, PINK1 directly phosphorylates and ubiquitinates Parkin at serine 65 (Ser65) [164,165] and these modifications are necessary to ensure maximal recruitment and activation of Parkin at mitochondria [166,167,168]. Once recruited on mitochondria, Parkin ubiquitinates several mitochondrial outer membrane proteins in order to mediate the subsequent sequestration of mitochondria into the isolation membrane via the interaction with adaptor proteins [169]. The ubiquitin-binding adaptor protein p62/SQSTRM1 accumulates on depolarized mitochondria and, thanks to binding to LC3, facilitates recruitment of damaged mitochondria to autophagosomes [170].

Rab7 is fundamental also for mitophagy as, together with the TBC1D15/TBC1D17 RabGAP and Fis1, it functions as a mitophagy effector downstream of Parkin [29]. TBC1D15/17 belong to the TBC (Tre-2/Bub2/Cdc16) family with Rab-GAP functions [171,172], while Fis1 is a fission protein having a cytosolic N-terminal domain and being anchored to the mitochondrial outer membrane with its C-terminal domain [173,174]. In the absence of TBC1D15, or with TBC1D15 lacking Rab-GAP activity, LC3-labeled isolation membranes accumulate excessively and without cargo orientation, sending long membrane tubules away from mitochondria along microtubule tracks [29]. Thus, the authors suggest that, during mitophagy, TBC1D15 binds LC3 and Fis1 to coordinate Rab7 activity, in order to shape the nascent autophagosome isolation membrane [29]. In fact, Rab7 activity, initially finalized to promote autophagosomal membrane growth and microtubule associated trafficking, could be then tempered by TBC1D15/17 Rab-GAP activity in order to tailor autophagosomal membrane expansion, so that it can correctly surround mitochondria [29] (Figure 3B). Indeed, Rab7 silencing suppresses the abnormal LC3 accumulation and tubulation in TBC1D15 -/- cells [29]. These data indicate that, in case of Parkin regulated mitophagy, at variance with general macroautophagy, Rab7 could be important for the expansion of the LC3-positive isolation membrane, and that termination of Rab7 activity could be required to mediate the release of LC3-bound membranes from microtubules, as they contact the mitochondrial cargo [29,70]. This model differs substantially from the established role of Rab7 in controlling the final step of maturation of autophagosomes accomplished by fusion with lysosomes [138,139]. In addition, the interaction between the mitochondrial fusion-related protein Mitofusin 2 (MFN2) and Rab7 significantly increases in response to starvation, suggesting the involvement of Rab7 as adaptor protein used by MNF2 during maturation of the autophagosomal membrane [175]. Thus, in mitophagy, Rab7 has a role both in autophagosome formation and autophagosome maturation.

### 3.3. Role of Rab7 in Lipophagy 

The balance between storage and catabolism of lipids has a central role in the liver with important implications in several pathologies, such as steatosis. Lipid droplets (LDs), composed by triacylglycerol (TG) and cholesteryl esters, are stored in hepatocytes that catabolize LDs mainly through autophagy, better known as lipophagy [176]. In this process, LDs are engulfed by an autophagosome, which then mature into an autolysosome to allow acid lipases to break down TG in its glycerol and fatty acid components. Inhibition of the lipophagy pathway in cells and mice leads to hepatocellular steatosis [177]. 

Rab7 is important in the regulation of lipophagy as it has been suggested that it promotes “synapse” formation between LDs and autophagosomes [178]. In fact, Rab7 is a fundamental component of LDs and its recruitment and activation are necessary for autophagic LD catabolism under nutritional condition stress. Interestingly, LD-associated Rab7 drives the fusion of LDs with MVBs and late endosomes prior to lysosomal fusion [178]. Thus, Rab7 is a key player in the regulation of targeting and fusion of “primed” autophagic LDs to late endocytic compartments such as late endosomes and lysosomes in order to produce energy (Figure 3C).

## 4. Rab7 and the Cytoskeleton 

As discussed above, Rab7, by recruiting effector proteins such as RILP and FYCO, is able to anchor Rab7-positive vesicles and compartments to microtubule motor proteins such as kinesin and the dynein–dynactin complex, in order to regulate organelles movements on both directions on microtubule tracks. However, Rab7 seems also to have direct roles in the regulation of the other two components of the cytoskeleton: intermediate filaments and actin microfilaments.

Intermediate filaments (IFs) are insoluble protein polymers that are assembled from soluble precursors [179] and constitute one of the three components of cytoskeleton [180]. IFs are the major determinants of cell architecture, but they have also many other functions such as, for instance, the regulation of membrane trafficking and of organelle positioning and function [181,182,183,184]. Indeed, several types of organelles, such as nucleus, mitochondria, the Golgi apparatus, endosomes and lysosomes, interact with IFs [185,186]. Furthermore, a number of IFs proteins interact with vesicular trafficking regulatory proteins [187]. For instance, vimentin and peripherin bind to complex adaptor protein AP3 that is involved in transport between endosomes and lysosomes and their distribution and positioning are altered as consequence of changes in the IF network with consequent dysfunctions [188]. Peripherin and vimentin also directly interact with Rab7 [189,190]. In Rab7-silenced cells, the amount of insoluble (filamentous) peripherin and vimentin increases while expression of the constitutively active Rab7 Q67L mutant augments soluble vimentin and peripherin, thus inducing their disassembly [189,190]. These data indicate that Rab7 is fundamental for assembly of some kind of intermediate filaments, suggesting that it could influences several cellular processes controlled by these intermediate filaments [187].

A connection between Rab7 and the actin cytoskeleton was also recently discovered [191,192,193]. First of all Rab7 interacts with Rac1, a small GTPase involved in the regulation of actin cytoskeleton [194]. In addition, a new effector of Rac1 called Armus was identified [193]. Armus is a TBC Rab-GAP that inactivates Rab7 coordinating Rab7 and Rac 1 functions during autophagy [192]. In addition, it was demonstrated that overexpression of Rab7 increased Rac1 activity while Rab7 silencing caused Rac1 inactivation [191]. Furthermore, it was recently demonstrated that Rab7 colocalizes with cortactin and f-actin in circular dorsal ruffles, membrane protrusions composed of actin-rich structures, and that overexpression of Rab7 induces their formation [191]. These data indicate that Rab7 is important also for actin cytoskeleton organization.

## 5. Rab7 Implications in Apoptotic Response: Debated Role as Tumor Suppressor

In the past years, a role of Rab7 as pro-apoptotic factor emerged [25]. Silencing of Rab7 or expression of a dominant-negative Rab7 mutant induce survival independence from nutrient uptake, through blocking glucose and amino acid proteins transporter degradation, and abrogating atrophic sequelae of changes that characterize growth factor withdrawal [25]. Furthermore, Rab7 inhibition is accompanied by maintenance of phosphorylation of the eukaryotic initiation factor 4E-Binding protein 1 (EIF4EBP1) activating cap-dependent RNA translation [25]. Moreover growth factor availability regulates Rab7 activity as, during growth factor deprivation, activation of Rab7 on lysosomal membranes prompts apoptosis determining reversion of growth factor independent survival in cells deficient of pro-apoptotic factor PKCδ [195]. The pro-apoptotic role of Rab7 leads to the hypothesis of a direct correlation with tumor proliferation independent of growth factor survival [196]. Indeed, Rab7 depletion promotes transformation of mouse embryo fibroblasts lacking p53 and expressing adenovirus E1A protein [25].

The specific role of Rab7 in cancerous events, however, is still not completely clear. In the literature, both pro-tumorigenic [197,198,199] and oncosuppressor functions [25,32,200] of Rab7 are supported [199], as Rab7 could have dose- and tumor-type-dependent roles in cancer cell proliferation and invasion. Indeed, changes of Rab7 expression levels were described in melanoma cells and tumor specimens [199]. In particular, it was demonstrated that low levels of Rab7 are maintained in benign nevi, while during melanoma development, the oncogenic transcription factor myc is activated and induces a strong overexpression of Rab7 [199]. Subsequent selective downregulation of Rab7 expression is associated with cancer progression in order to favor the invasive phenotype and to switch to metastatic stages [199]. In *C. elegans*, knockdown of Rab7 mimicked p53-independent apoptosis thus revealing its anti-apoptotic function [201].

In a recent work, a correlation between tumor cell invasion and anterograde movement of lysosomes as consequence of tumor microenvironment stimuli was discovered [32]. In particular, it was demonstrated that acid extracellular pH induces lysosomal movement toward to cellular periphery and successive lysosomal exocytosis of cathepsin B, promoting protease-dependent tumor invasion [202,203]. Troglitazone is a peroxisome proliferator-activated receptor-γ (PPAR-γ) agonist, used for the treatment of type II diabetes, because of its ability to improve insulin sensitivity. This compound has several PPAR-γ-independent effects and, for instance, influences cell migration and invasion in several malignancies [204]. Observation that troglitazone prevents invasion in prostate cancer cell after extracellular acid pH stimuli, lead to the demonstration that Rab7 is the negative regulator of cell surface-directed lysosome trafficking, determining abrogation of cathepsin B secretion and tumor cell invasion [32]. This evidence was further sustained by demonstration that Rab7-silenced cells exhibited increased levels of secreted proteases and were more invasive in vitro [200,205] while tumors derived from these cells grow faster and exhibit an increased invasive phenotype in vivo [32].

In addition, Rab7 knockdown in prostate cancer cells caused high levels of c-Met, a protein involved in the signaling axis for the regulation of cell invasion and metastasis [206]. Thus, Rab7 could be considered a negative regulator of many pro-survival signals from the cell surface. In fact, the Rab7 effector Rabring7 is an E3 ligase involved in EGFR degradation and kinetics of EGFR degradation are affected by expression of Rab7 dominant negative mutant [76,207]. In addition, stability of EGFR and Her2 and sustaining of the subsequent survival signaling were guaranteed by Rab7 [197]. In fact, a synergic action between Rab7 depletion and HSP90 inhibition reduces the level of EGFR and Her2 through proteasomal degradation and promotes apoptosis counteracting previous theory about pro-apoptotic action of the Rab7 GTPase. 

Interestingly, it was recently demonstrated that PTEN, a phosphatase that acts on both lipid and protein substrates [208], suppresses EGFR-mediated cell growth and proliferation signaling [209]. PTEN is able to convert phosphatidylinositol-3,4,5-trisphosphate (PIP3) to phosphatidylinositol-4,5-bisphosphate (PIP2) at the cellular membrane, negatively regulates oncogenic PI3K-AKT signaling and, recently, it was shown that is associated with PI(3)P-containing endosomes [210]. Rab7 S72 and Y183 residues are crucial for Rab7 association with GDI, subsequent Rab7 delivery to late endosomal membranes and for activation by the Mon1a–Ccz1 GEF complex, which in turn is required for maturation of late endosomes. The recently discovered PTEN-dependent regulation of Rab7 strengthens the link between Rab7 and cancer [211]. Thus, PTEN acting on Rab7, provides alternative mechanism for spatial and temporal control of EGFR signaling through activation of Rab7-mediated endosome maturation [211]. Notably, mutation of PTEN at residue 138 occurs in several tumor [212,213] and inactivates PTEN with loss of the control of Rab7-dependent endosomal degradation of EGFR and consequent uninterrupted growth signaling with important implications for tumor progression [211]. 

In this complex picture, the different functions of Rab7 probably depend on cellular context and on other environmental factors. For instance, when survival of cells depends on nutrient transporters Rab7 may act as a pro-apoptotic factor because of its role in endocytic traffic to promote lysosomal degradation of the transporters [25], while in cells that are more dependent on surface growth factor receptors, such as EGFR and Her2, Rab7 may act as pro-survival proteins protecting EGFR and Her from proteosomal degradation [197].

## 6. Role of Rab7 in Specialized Cells 

### 6.1. Role of Rab7 in Neurons

**Figure 4:**
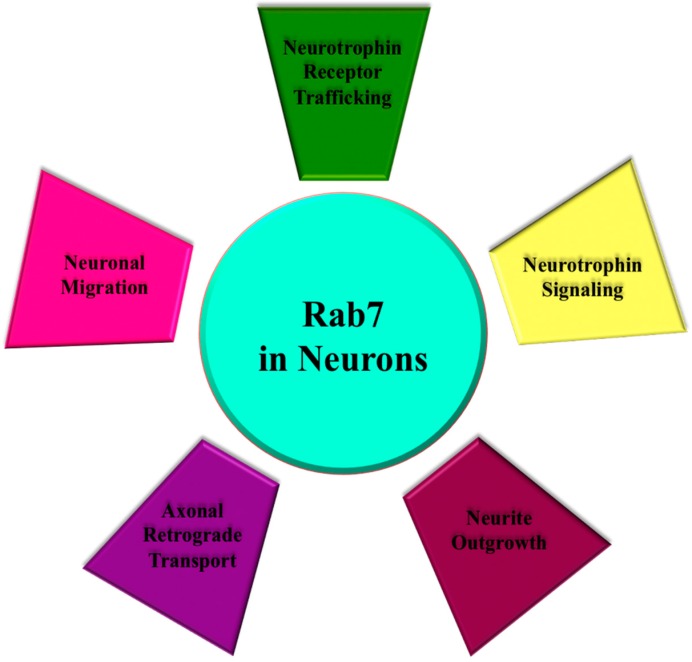
Multiple roles of Rab7 in Neurons. Rab7 has a crucial role in different neuronal processes.

Rab7 is a ubiquitous protein with a key role in the endocytic pathway but it has also specific functions in neurons (Figure 4).

In fact, Rab7 interacts with the nerve growth factor (NGF) receptor TrkA (Tropomyosin receptor kinase A) and controls TrkA endosomal trafficking and signaling [26,27]. Expression of a Rab7 dominant negative mutant in NGF stimulated PC12 cells determines endosomal accumulation of TrkA and augmented TrkA signaling, leading to the increase of downstream effects, such as phosphorylation of Erk1/2, up-regulation of growth associated protein 43 (GAP43) and strongly enhancing neurite outgrowth in response to limited NGF stimulations [26]. The role of Rab7 in neurite outgrowth was also recently confirmed in a recent report where a new effector of Rab7, protrudin, has been identified [214]. Protrudin is an endoplasmic reticulum protein that promotes protrusions and neurite outgrowth [215]. The direct interaction between Rab7 and protrudin allows endoplasmic reticulum to form contact sites with late endosomes [214]. These contacts determine transfer of kinesin 1 from protrudin to FYCO1 on late endosomes to promote translocation of late endosomes to the cell periphery and subsequent synaptotagmin-VII-dependent fusion with the plasma membrane that is required for outgrowth of protrusions and neuritis [214]. 

Rab7 is also important for the regulation of the biogenesis and progression of axonal retrograde transport carriers in motor neurons, acting in concert with Rab5 to guarantee the integrity of the axonal transport machinery, essential for neuronal survival [27]. In particular, it was shown that Rab5 and Rab7 sequential activities are required for coupling specialized clathrin-dependent endocytosis to fast retrograde axonal transport [27]. Using a fragment of tetanus neurotoxin, which bind with high affinity motor neurons and is endocytosed in clathrin-coated vesicles, it was shown that Rab5 is responsible for the early sorting of the tetanus neurotoxin, while Rab7 guarantees long-range axonal transport, through the control of later events and by ensuring the movement of tetanus toxin containing organelles [27]. Thus, Rab5 was associated with stationary or oscillatory organelles while Rab7 was localized with a subpopulation of moving compartments and impairment of Rab7 function leads to a complete blockade of axonal transport [27].

Neuronal migration is regulated by several endocytic Rabs [216]. In fact, the migration of immature neurons during the development of cerebral cortex in its specific layered structure is regulated by Rab5, Rab7 and Rab11 [216]. In particular, Rab7, by controlling the late endocytic pathway, seems to influence the final phase of neuronal migration being important for dendrite morphology [216].

Notably, mutations in the Rab7 gene cause the Charcot-Marie-Tooth type 2B (CMT2B) peripheral neuropathy [33,34,217]. Although the exact mechanism underlying this pathology is not yet fully understood, the fact that mutations in a ubiquitous protein such as Rab7 affect only peripheral neurons can be explained considering the neuronal specific functions of Rab7. In fact, four CMT2B-associated Rab7 mutants, which display altered GTP binding properties compared to wild-type [218,219,220], inhibit neurite outgrowth in several different cell lines [221,222]. Expression of these Rab7 mutants affected the outgrowth of long neuritis (more than 50 micron in length) suggesting that altered lysosomal biogenesis, degradation of nutritional factors and their transporters and/or altered endosomal signaling impact on neurite outgrowth [221]. 

The recently discovered interaction of Rab7 with two intermediate filament proteins, vimentin and peripherin, that have specific roles in neurons, could help to explain the molecular basis of CMT2B, considering that CMT2B-causing Rab7 mutant proteins display altered interactions with these two intermediate filament proteins [189,190]. Although mature neurons normally not express or express vimentin at very low levels, vimentin is strongly expressed during axonal regeneration after injury [223]. Peripherin has role in neuritogenesis, axonal outgrowth and axonal regeneration and it is considered a neuronal differentiation marker, induced by stimulation with NGF [224,225,226,227,228,229,230]. Interestingly, during aging, overexpression of peripherin causes degeneration of motor axons leading neuron dysfunction and the slowing down of neurofilament protein transport [231,232,233]. In light of this, variations of vimentin/peripherin distribution and function, due to the expression of the CMT2B-causing Rab7 mutant proteins, can impair the damage-response program, affect neurofilament dynamics and, as a consequence, cause axonal degeneration characteristic of this pathology [189,190].

### 6.2. Role of Rab7 in Osteoclasts

Osteoclasts are specialized bone cells that degrade the bone matrix. Activated osteoclasts present a convoluted ruffled border membrane where the majority of Rab7 is localized [234]. Thus, in contrast to other cell types where Rab7 localization is restricted to late endosomes and lysosomes, in osteoclasts Rab7 localizes mainly at specific domains (the ruffled border) of the plasma membrane. In these cells, Rab7 regulates osteoclast polarization and, importantly, bone matrix resorption [235]. Thus, Rab7 is responsible for the secretion in the extracellular space of lysosomal enzymes that take care of bone matrix degradation. In addition, it has been proposed that the formation of osteoclast ruffled border is regulated by the Rab7–Rac1 interaction [194].

## 7. Other Roles of Rab7

Rab7 seems to have some other functions although less exploited. For instance, it has been involved, together with Rab27, in the regulation of the secretion of endothelial miRNA through extracellular vesicles [236]. 

Rab proteins regulate trafficking also of membrane channels and Rab7, in particular, has been found to affect KCNQ1/KCNE1 potassium channels [237,238]. In fact, expression of the Rab7 dominant negative mutant causes an increase of plasma membrane localization of mutated channels associated with the long QT syndrome, increasing channel-mediated currents [238]. Furthermore, a number of mutated potassium channels were found to colocalize with Rab7 on late endosomes [238]. These data suggest that disease-associated mutant KCNQ1/KCNE1 potassium channels are stored in late endosomes and their trafficking is controlled by Rab7.

Rab7 is also important for the mechanism of HBV (Hepatitis B Virus) secretion. Indeed, it was found that increased Rab7 activation induces dramatic changes in the morphology of MVB and autophagic compartments, characterized by the formation of numerous tubules, strongly altering HBV secretion [239].

Moreover, Rab7 is responsible for diminished opioid responsiveness of peripheral sensory neuron μ-opioid receptors (MORs) in rats with streptozotocin-induced diabetes and this fact has important implications in painful diabetic neuropathy [240].

Finally, in plants, the understanding of the role of Rab7 is still limited but a recent work demonstrates that Rab7 overexpression in rice enhances tolerance to salt stress [241].

## 8. Conclusions

In this review, we discussed the main functions of the small GTPase Rab7 highlighting that the central role of Rab7 for correct cargo selection, biogenesis, positioning, motility and functioning of lysosomes, phagolysosomes and autolysosomes is fundamental for a number of other cellular processes. In fact, as Rab7 is responsible for trafficking and degradation of several molecules, including signaling receptors and adhesion molecules as well as of different organelles, it takes part in several cell mechanisms governing cellular homeostasis. Therefore, it is now clear that Rab7 is a central molecule of the cell, playing key roles also in cell survival, differentiation and apoptosis, although its exact molecular mechanism of action in these processes has still to be investigated in details. Importantly, currently available data on Rab7 and diseases suggest that modulation of Rab7 expression and activity could help to counteract a number of pathologies, such as Charcot-Marie-Tooth type 2B and cancer. Further work will be necessary to investigate this possibility.

## Abbreviations

The following abbreviations are used in this manuscript:

ALDH2	Aldehyde dehydrogenase 2
ALR	Autophagic Lysosome Reformation
ARF6	ADP-ribosylation factor 6
ATG	Autophagy-related gene
CI-M6PR	Cation-Independent Mannose 6-Phosphate Receptor
CMT	Charcot-Marie-Tooth
CMT2B	Charcot-Marie-Tooth type 2B
EGFR	Epidermal Growth Factor Receptor
ESCRT	Endosomal Sorting Complex Required for Transport
EIF4EBP1	Eukaryotic Initiation Factor 4E-Binding protein1 (EIF4EBP1)
FYCO	FYVE and COiled-coil domain containing protein
GAP	GTPase Activating Protein
GDI	GDP Dissociation Inhibitor
HBV	Hepatitis B Virus
HOPS	HOmotypic fusion and Protein Sorting
Ifs	Intermediate filaments
IGF-I	Insulin-like Growth Factor-1
LAMP-1	Lysosomal-Associated Membrane Protein 1
LAMP-2	Lysosomal-Associated Membrane Protein 2
LBPA	LysoBisPhosphatidic Acid
LD	Lipid Droplet
LDL	Low-Density Lipoproteins
LC3	microtubule-associated protein 1A/1B-Light Chain 3
MFN2	Mitofusin 2
MORs	μ-Opioid Receptors
MVBs	MultiVesicular Bodies
MTOC	MicroTubule-Organizing Center
mTORC1	mammalian Target Of Rapamycin Complex 1
NGF	Nerve Growth Factor
ORP1L	Oxysterol-binding protein-related Protein 1 L
PI3P	PhosphatidylInositol-3-Phosphate
PI3K	PhosphatidylInositol-3 Kinase
PKC	Protein Kinase C
PINK1	PTEN-Induced Kinase 1
PTEN	Phosphatase and TENsin homolog
RILP	Rab-Interacting Lysosomal Protein
SNARE	SNAP (Soluble NSF Attachment Protein) REceptor
SNX	Sorting NeXin
TBC	Tre-2/Bub2/Cdc16
TGN	Trans-Golgi Network
TrkA	Tropomyosin receptor kinase A
V-ATPase	Vacuolar ATPase
VSV	Vesicular Stomatitis Virus
